# A Case of an Infected Right Ventricular Pseudo-False Aneurysm

**DOI:** 10.7759/cureus.92999

**Published:** 2025-09-23

**Authors:** Yuki Hayashi, Atsushi Harada, Fumihiro Kitashima, Hiroyuki Hao, Masashi Tanaka

**Affiliations:** 1 Department of Cardiovascular Surgery, Nihon University School of Medicine, Tokyo, JPN; 2 Division of Human Pathology, Department of Pathology and Microbiology, Nihon University School of Medicine, Tokyo, JPN

**Keywords:** false aneurysm, infected pseudo-false aneurysm, pseudo-false aneurysm, right ventricular pseudoaneurysm, right ventricular pseudo-false aneurysm

## Abstract

A pseudo-false aneurysm is a rare complication of a myocardial infarction. This report describes our surgical experience with an extremely rare case of an infected right ventricular (RV) pseudo-false aneurysm. A 79-year-old woman with a history of steroid use for rheumatoid arthritis who was immobile for a few days presented to our hospital. She was in septic shock, so she was admitted to a critical care center, where antibiotics were initiated. Electrocardiography at the time of admission suggested an inferior wall myocardial infarction; however, given the patient’s general condition, she was treated conservatively. After resolution of sepsis, transthoracic echocardiography and computed tomography revealed a mass shadow that suggested an RV pseudoaneurysm. Therefore, we performed pseudoaneurysm repair and coronary artery bypass of the left anterior descending branch. On opening the chest, we noted that the mass exhibited no adhesions to the pericardium or hematomas. The mass wall comprised firm tissue, while fragile tissue was observed inside. A pathological examination of the mass wall revealed myocardial tissue and Gram-positive cocci, with no findings suggestive of a myocardial infarction. Therefore, an infected RV pseudoaneurysm was diagnosed. The patient had an uneventful postoperative course and was discharged on postoperative day 40. Few reports of pseudo-false aneurysms are available, and most have described pseudoaneurysms in the left ventricle. To our knowledge, no reports have described pseudoaneurysms in the right ventricle. Therefore, cases like ours, which are associated with infection, are considered very rare.

## Introduction

A pseudo-false aneurysm is a very rare complication of a myocardial infarction (MI). Unlike pseudoaneurysms (also called false aneurysms), which are caused by a completed infarction, pseudo-false aneurysms are formed in the myocardial layer as a result of an uncompleted laceration of the infarcted myocardium [1]. Pseudo-false aneurysms are characterized by the absence of adhesion to the pericardium, which is different from pseudoaneurysm [1]. Most pseudo-false aneurysms originate from the left ventricle. It is essentially a complication of MI and occurs almost exclusively in the left ventricle. To our knowledge, no pseudo-false aneurysms with a right ventricular (RV) origin have been reported. Additionally, 5% of pseudoaneurysms have an infectious origin [2]; however, to our knowledge, no reports have described pseudo-false aneurysms that originated from infections. Whether it is a pseudoaneurysm or a pseudo-false aneurysm, there is a risk of rupture. Therefore, surgical intervention is required. We present a very rare case where early surgery was performed for the purpose of preventing rupture based on suspected ventricular pseudoaneurysm on imaging studies, only to reveal it was a pseudo-false aneurysm.

## Case presentation

A 79-year-old woman with a history of steroid use (prednisone 7.5 mg daily) for rheumatoid arthritis experienced difficulty moving for a few days and was transported to our hospital. Electrocardiography revealed ST elevation in leads Ⅱ and Ⅲ and aVF. Laboratory tests revealed the following findings: white blood cell count, 18,800/μL; neutrophil fraction, 97%; C-reactive protein level, 32.6 mg/dL; creatine kinase level, 503 U/L; creatine kinase-MB level, 33 U/L; and troponin I level, 0.67 ng/mL (Table 1).

**Table 1:** Laboratory investigations

Parameters	At admission	Reference values
White blood cell count (/μL)	18,800	4000-10,000
Neutrophil fraction (%)	97	42-75
C-reactive protein (mg/dL)	32.6	<0.30
Creatine kinase (U/L)	503	56-244
Creatine kinase-MB (U/L)	33	<25
Troponin I (ng/mL)	0.67	<0.04

**Figure 1:**
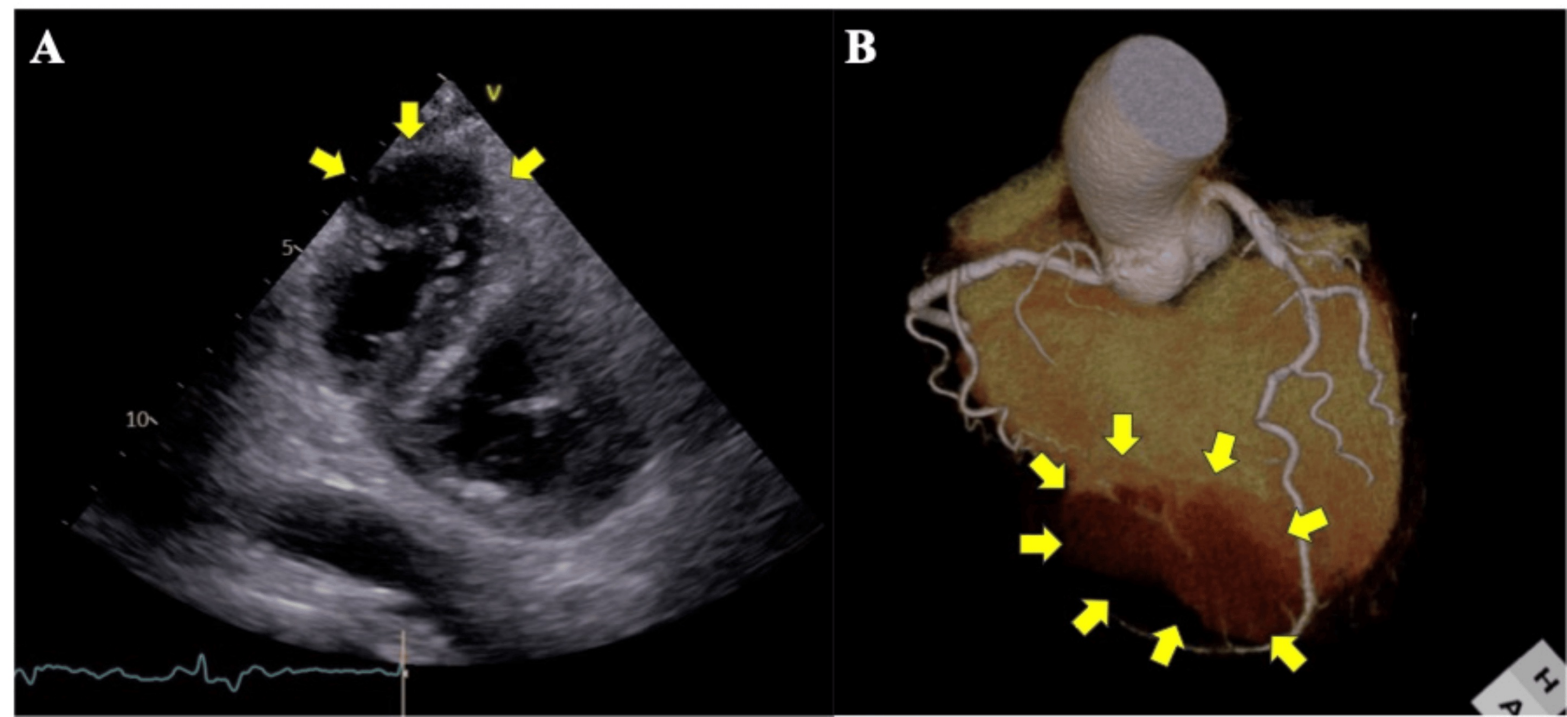
Preoperative transthoracic echocardiography (A) and enhanced three-dimensional computed tomography (B) images. A: Left ventricular short-axis view showing a tumor shadow continuous with the right ventricle (arrow). B: Left anterior descending branch with 75% stenosis and a pseudo-false aneurysm (arrow).

Septic shock was diagnosed, and supplemental fluids, catecholamines, and antibiotics were initiated. Electrocardiography findings suggested an MI in the inferior wall; however, based on the patient’s general condition, conservative management and follow-up of myocardial enzyme levels were conducted. Blood culture results did not indicate any bacteria; however, the sputum culture results indicated *Staphylococcus aureus*. Pneumonia and dehydration were considered causes of the septic shock, which rapidly resolved a few days after treatment. Subsequently, transthoracic echocardiography and coronary enhanced computed tomography (CT) were performed. Transthoracic echocardiography revealed an RV apex perforation and pseudoaneurysm (Figure 1A), with blood flow from the right ventricle into the aneurysm, as well as severely reduced left ventricular inferior wall motion with a normal ejection fraction.

**Figure 2:**
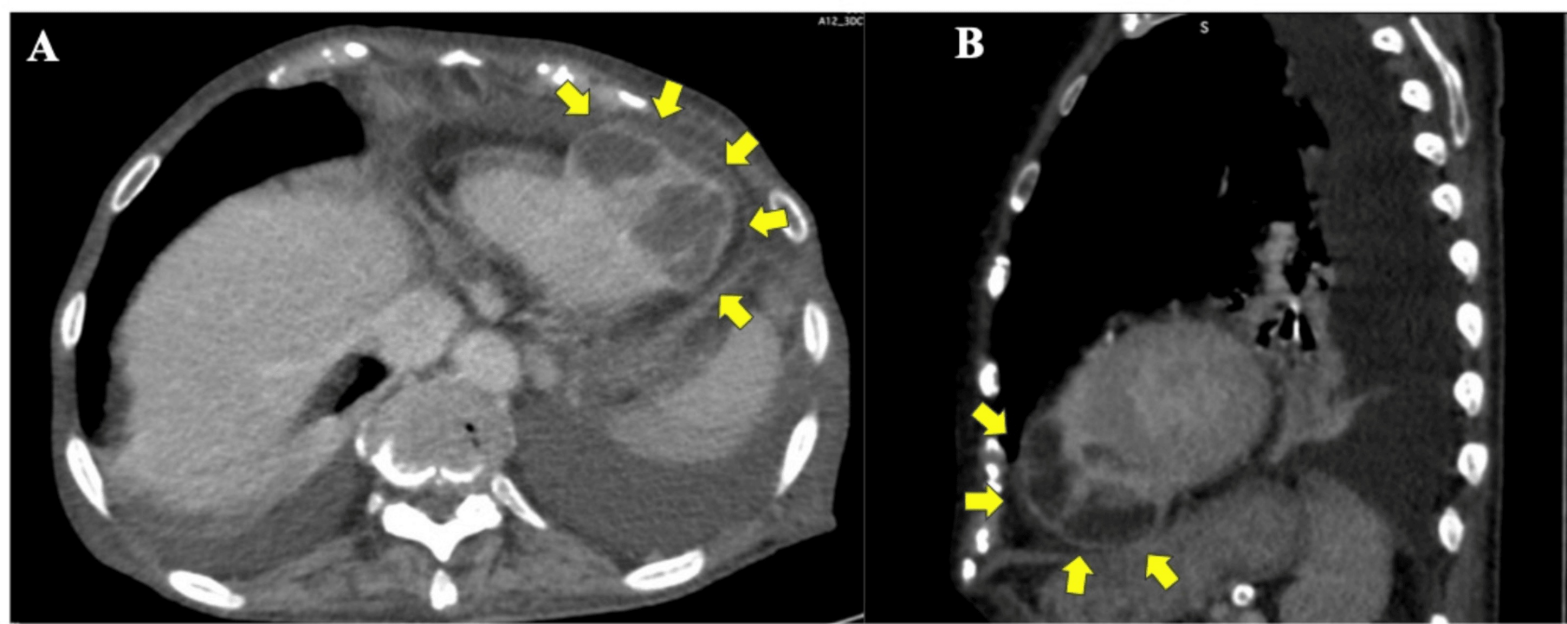
Enhanced computed tomography images (A: horizontal section; B: sagittal section). A 53-mm × 40-mm tumor with contrast enhancement around its periphery is connected to the right ventricle (arrow).

Coronary enhanced CT revealed 75% stenosis in the proximal left anterior descending (LAD) artery, a perfusion zone beyond the apex of the heart (Figure 1B), and a 53-mm × 40-mm aneurysm on the RV posterior wall (Figure 2).

An RV pseudoaneurysm with impending rupture after a subacute MI was diagnosed; therefore, immediate surgery was planned.

**Figure 3:**
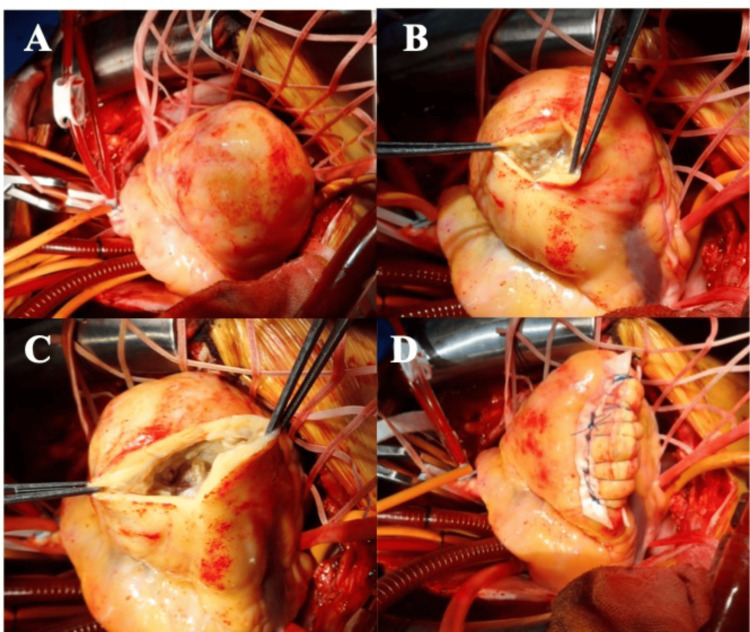
Surgical findings of the head (left) and tail (right). A: The anterior wall of the right ventricle shows no adhesion to the pericardium, and no pseudotumor is visible on the surface. B and C: Necrotic myocardium is observed upon incision of the epicardium, and no internal hemorrhage is noted. D: Linear closure is performed by removing the pseudotumor as much as possible.

Median sternotomy was performed under general anesthesia. Incision of the pericardium revealed pale yellow, turbid pericardial fluid without bleeding. No adhesions were observed between the pericardium and epicardium, and the myocardium around the ventricular mass comprised solid tissue without hematomas (Figure 3A).

**Figure 4:**
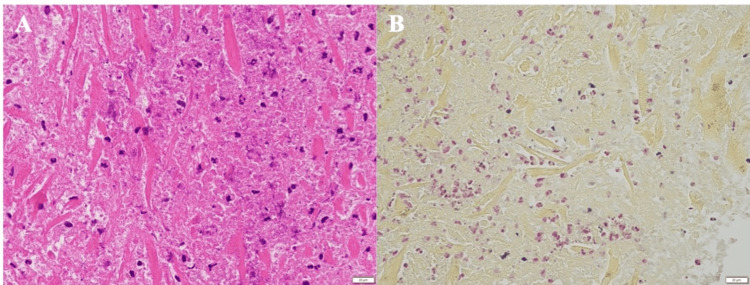
Pathological findings. A: Hematoxylin and eosin staining. Necrotic myocardium, residual myocardium, and numerous neutrophil infiltrates are present. No typical signs of MI in the necrotic myocardium are apparent. These findings strongly suggest necrosis attributable to bacterial infection. B: Gram staining. Neutrophil infiltration accompanied by a small amount of Gram-positive bacteria. The bacterial type could not be identified microscopically.

Cardiac arrest via cardioplegia was established, cardiopulmonary bypass was performed, and the RV aneurysm was opened. Milky fluid retention and fragile myocardium were noted; however, no obvious blood flow from the RV cavity was observed (Figures 3B-3C). The fragile tissue of the RV mass was debrided, and linear closure was performed (Figure 3D). Finally, coronary artery bypass from the ascending aorta to the LAD artery using the great saphenous vein was performed. The cardiopulmonary bypass time was 110 minutes, the cross-clamp time was 74 minutes, and the operative time was 239 minutes. Postoperative transthoracic echocardiography and enhanced CT revealed that the RV mass had disappeared. A pathological examination revealed residual myocardium and findings suggestive of a pseudoaneurysm. Furthermore, although myocardial necrosis was present, the typical findings of MI, namely, a clearly defined border between the necrotic area and the normal myocardium, were not observed. Numerous Gram-positive necrotic cells and neutrophil infiltration were observed, which strongly suggested an MI (Figure 4).

The patient’s postoperative course was uneventful, and she was discharged on postoperative day 40.

## Discussion

A ventricular pseudoaneurysm is an uncommon complication after an MI, with an incidence of 0.2% to 0.3% [3]. Such pseudoaneurysms occur after hemorrhagic dissection penetrates the MI area. Furthermore, a pseudoaneurysm is an intramyocardial dissection that extends to the epicardium, and the epicardium becomes an aneurysm as a result of adhesion between the epicardium and pericardium without myocardial tissue in the wall [4]. Most ventricular pseudoaneurysms originate from the left ventricle and rarely occur in the right ventricle. RV pseudoaneurysms caused by iatrogenic or traumatic events have been reported [4-6]; however, they rarely occur after MI [7]. A pseudo-false aneurysm occurs when a hematoma that forms after an MI does not reach the epicardium and is confined to the myocardium [4]. Additionally, a pseudo-false aneurysm is rarer than a pseudoaneurysm. The risk of rupture of an untreated pseudoaneurysm is approximately 40% [2]. Consistent reports of pseudo-false aneurysms are not available; therefore, the incidence and probability of their rupture are unknown. Surgical removal is the preferred treatment for pseudoaneurysms because of the risk of rupture.

Most reported cases of pseudo-false aneurysms have been observed in the inferior, apical, and posterior walls of the left ventricle; however, rare cases in the septum and anterior wall have been reported [8,9]. A few cases of left ventricular pseudo-false aneurysms that perforated the right ventricle have been reported as well [10,11].

MI is the most common cause of ventricular pseudoaneurysms; however, rare cases have been caused by infection. Most cases of pseudoaneurysms caused by infection are associated with infective endocarditis [12,13]; however, to our knowledge, no cases of pseudo-false aneurysms associated with infection have been reported.

The present case was diagnosed as a pseudo-false aneurysm based on the absence of intraoperative adhesions between the pericardium and epicardium and pathology results that revealed the myocardium in the mass wall. The cause of our patient’s pseudoaneurysm was initially suspected to be an MI; however, the pathological findings did not reveal any signs of MI. Instead, significant neutrophil infiltration and Gram-positive bacteria were observed, which led to the conclusion that infection was the most likely cause. Preoperative coronary CT revealed 75% stenosis of the LAD artery without occlusive lesions, suggesting relative ischemia of the LAD artery attributable to dehydration caused by infection. However, it is possible that the infection did not lead to an MI. The patient had a long history of oral prednisone use and was prone to infection. *Staphylococcus aureus* was detected by the sputum culture and considered the possible pathogen in this case; however, it was not detected by the blood culture. Infectious endocarditis was ruled out. Therefore, we considered that our patient experienced a very rare condition comprising pseudo-false aneurysm development caused by MI, which may have led to infection of the necrotic myocardium. However, this diagnosis was ruled out by the pathological examination. Therefore, based on the pathological findings, this case was considered to have resulted in infection-related myocardial necrosis rather than MI, leading to pseudo-false aneurysm formation.

## Conclusions

The presence of an infection in pseudo-false aneurysm tissue is rare. We believe that such a case in the right ventricle alone is unprecedented; however, we expect to accumulate more cases in the future. We encountered an unusual case of an infected pseudo-false aneurysm in the right ventricle. A pseudo-false aneurysm cannot be diagnosed until the chest is opened.
